# Cognitive-behavioral phenotypes in *FMR1* premutation carriers: a cross-population comparison with mild cognitive impairment

**DOI:** 10.3389/fnmol.2026.1943587

**Published:** 2026-09-10

**Authors:** Nell Maltman, Jessica Greenlee, Kimberly D. Mueller, Barbara B. Fransway, James T. Shira, Davina Hammann, Sumi Lee, Hyeri Lee, Ali Naderi Malek

**Affiliations:** 1 Department of Speech, Language, and Hearing Sciences, University of Arizona, Tucson, AZ, United States; 2 Department of Psychology, Lafayette College, Easton, PA, United States; 3 Department of Communication Sciences and Disorders, University of Wisconsin-Madison, Madison, WI, United States; 4 Arizona Genetics Core, University of Arizona, Tucson, AZ, United States

**Keywords:** *APOE*, cognition, depression, *FMR1*, fragile X premutation, language, mild cognitive impairment

## Abstract

**Introduction:**

Carriers of the *FMR1* premutation are at risk for cognitive decline, though premutation-specific markers of age-related risk are largely unknown. One approach to determining relevant risk markers is to assess cross-population comparisons with individuals with known clinical trajectories.

**Methods:**

The present study evaluated a cross-sectional sample of male and female *FMR1* premutation carriers and controls in comparison to a longitudinal sample of individuals with known progression to mild cognitive impairment (MCI) within an enriched cohort of individuals at risk for Alzheimer’s Disease and Related Dementias (ADRD) to assess potential similarities across cognitive-behavioral and genetic features. Both participant samples completed measures of working memory (digit span), language (verbal fluency, narrative elicitation), and depression. Exploratory analyses evaluated the role of *FMR1* CGG repeat length and *APOE* on cognitive-linguistic phenotypes.

**Results:**

Findings suggest that premutation carriers and controls largely differed from MCI groups across measures; however, a subgroup of premutation carriers (according to T-MoCA performance) evidenced a distinct profile characterized by differences in depression and language. No effects of CGG repeat length or *APOE* were observed in the premutation sample.

**Discussion:**

This study generates preliminary evidence for a possible risk profile of *FMR1* premutation carriers, with important implications for longitudinal studies evaluating the progression of cognitive decline.

## 1 Introduction

The *FMR1* premutation occurs in ~1 in 151 females and ~1 in 470 males in the United States, and about 16% of females and 40% of males over the age of 50 develop fragile X-associated tremor/ataxia syndrome (FXTAS) (Jacquemont et al., 2004; Tassone et al., 2012b; Maenner et al., 2013); however, who will go on to develop the condition is unknown. FXTAS is characterized by intention tremor, executive dysfunction, gait ataxia, and cerebellar and white matter atrophy, and ~50% of male FXTAS patients present with dementia (Seritan et al., 2008). To develop effective interventions for individuals with FXTAS, it is critical to understand early markers of decline. Emerging evidence suggests a subset of premutation carriers (PMCs) with FXTAS exhibit similar patterns of cognitive decline (Seritan et al., 2008), neuropathology (Tassone et al., 2012a), and genetic risk markers (Silva et al., 2013) as individuals with other neurodegenerative conditions, namely mild cognitive impairment (MCI) and Alzheimer’s Disease and Related Dementias (ADRDs). Connected language, executive functioning, depression, and genetic markers are hypothesized to represent potential predictors of MCI/ADRDs and FXTAS; however, few studies have directly compared these populations to understand areas of phenotypic overlap, particularly in the pre-disease stage. The goals of this study are to evaluate cross-population features of cognition, language, behavior, and genetics among PMCs and controls alongside a sample of individuals who are known to have progressed to MCI.

Prior work has hypothesized similar trajectories of FXTAS and MCI/ADRDs, suggesting that early markers of MCI may be evident among PMCs prior to FXTAS onset. One promising marker of disease progression is language production, as differences from controls are evident among both PMCs and individuals with MCI-ADRDs over time. Numerous studies of female PMCs without FXTAS have documented differences in language use in both structured and open-ended language sampling contexts, observed across semantic, syntactic, and pragmatic domains (Losh et al., 2012; Sterling et al., 2013; Klusek et al., 2018; Bredin-Oja et al., 2021; Klusek et al., 2022a; Maltman et al., 2022a, 2026; Friedman et al., 2026). In contrast, studies of language use in male PMCs are quite limited, which constrains our understanding of potential pre-clinical markers of decline. Most studies of language in male PMCs have evaluated highly structured single-word contexts, such as verbal fluency tasks, often presented in the context of dual-task paradigms (Moore et al., 2004; Kraan et al., 2013; Hocking et al., 2015, 2017; Wang et al., 2017). In one study that used open-ended language sampling, male and female PMCs with FXTAS symptoms did not differ, and language production was linked to FXTAS symptom severity (Maltman et al., 2024).

Extant literature has documented differences in language use among individuals with MCI and ADRDs, even in the pre-clinical stage. Such differences from healthy controls have been observed in lexical-semantic, syntactic, fluency, and pragmatic aspects of language in both structured and open-ended connected language samples, such as picture description and verbal fluency tasks (Araujo et al., 2011; Clark et al., 2014; Mueller et al., 2018b). To our knowledge, only one study has directly compared language between individuals with FXTAS and amnestic MCI, in which they evaluated verbal learning and N400 ERP responses (Yang et al., 2014). They found similarities between groups in their N400 responses, which differed from controls, reflecting possible shared impairments in semantic processing. Taken together, these findings suggest that language may be impacted in both male and female PMCs, with possible links to FXTAS, and which may reflect a promising marker of age-related decline.

Another candidate marker of FXTAS progression is executive functioning. Difficulties with executive functioning are common in male and female PMCs, and executive dysfunction is a key feature of FXTAS (Kogan and Cornish, 2010; Berry-Kravis and Hall, 2011; Klusek et al., 2020; Hocking et al., 2021; Maltman et al., 2021, 2022; Hessl et al., 2023). In a longitudinal study of FXTAS, Hessl et al. (2023) found that metacognitive features of executive functioning (e.g., working memory) predicted conversion to FXTAS among male PMCs, making it among the most promising predictive markers of the condition. Interestingly, in a study comparing FXTAS and AD, Seritan et al. (2008) identified no significant differences between populations on working memory (digit span backward), phonemic fluency (reflecting executive control), or naming between populations. This suggests that, at the disease stage, executive functioning may be similar; however, whether executive functioning is similar across populations prior to disease onset is not well understood.

Psychiatric symptoms have been posited as a component feature of both FXTAS and MCI/ADRDs. In the *FMR1* premutation, depression is under the broader umbrella of fragile X-associated neuropsychiatric disorders (FXAND(Hagerman et al., 2018)). Studies across the lifespan have found ~28–50% of PMCs have symptoms of depression (Bailey et al., 2008; Hagerman and Hagerman, 2016; Allen et al., 2020), impacted by age, CGG repeat length, and parenting stress (Roberts et al., 2016; Klusek et al., 2025). Depression symptoms may cluster with co-occurring conditions, such as headaches, neuropathy, tremor, ataxia, executive dysfunction, and FXTAS (Hocking et al., 2021, 2022; Flavell et al., 2023), and symptoms typically emerge in early adulthood, usually prior to cognitive-motor signs related to FXTAS (Bourgeois et al., 2026). In MCI/ADRDs, depression occurs in approximately half of cases and may represent a prodrome of the condition (Broe et al., 1990; Olin et al., 2002). For example, in the Framingham Heart Study, 21.6% of individuals with depression symptoms at baseline developed dementia as compared to only 16% of those without depression (Saczynski et al., 2010).

Beyond phenotypes, it is critical to note the extent of shared genetic contributions to disease onset and progression. *FMR1* CGG repeat length is a known contributor to cognitive-behavioral variability across the CGG range (up to 200 repeats). Increased CGG repeat length is thought to confer risk for FXTAS, with particular risk in the premutation mid-range of ~80–110 repeats (Hagerman and Hagerman, 2016). This range is hypothesized to contribute to an over-production of *FMR1* mRNA, causing a toxic gain-of-function that contributes to neurodegeneration (Gohel et al., 2020). Numerous studies have identified both linear and curvilinear effects of CGG repeat length, both within the premutation and in the general population, on executive functioning and cognition (Klusek et al., 2020, 2026; Mailick et al., 2020; Maltman et al., 2022b, 2023; Gabis et al., 2023; Tassone et al., 2026), language use (Klusek et al., 2018; Maltman et al., 2026), and depression (Roberts et al., 2009, 2016; Klusek et al., 2025).

Recent studies have assessed additional genetic modifiers to determine their potential role in the onset of FXTAS. Of particular interest is *APOE*, a genetic variant commonly implicated in the onset of ADRDs (Altmann et al., 2014; Burns et al., 2020; Montagne et al., 2020; Gharbi-Meliani et al., 2021). *APOE* has three allelic variations––*APOE* ε*2*, which is thought to contribute to cognitive resilience, *APOE* ε3, the most common and least phenotypically consequential, and *APOE* ε*4*, thought to confer to risk for ADRDs. In a study of males with FXTAS, Silva et al. (2013) found higher rates of the *APOE* ε4 allele relative to non-FXTAS PMCs, suggesting that this variant may confer additional susceptibility for the condition. Recently, Jiraanont et al. (2026) observed similar patterns, with additional findings that *APOE* ε2 appeared to play a protective role against neurodegeneration and cognitive impairments. Neither study, however, evaluated *APOE* expression prior to disease onset, limiting our understanding of genetic contributions to the phenotypic PMC spectrum.

Building on this prior literature, the present study examined cross-population comparisons of non-FXTAS *FMR1* PMCs and individuals known to progress to MCI (pre/MCI) on measures of cognition, language, and mood to better understand possible shared phenotypic overlap prior to disease onset, with additional evaluation into genetic contributions (*FMR1*, *APOE*). We asked the following questions:

1. To what extent do PMCs exhibit cognitive-behavioral similarities to individuals with pre/MCI?
2. Are there subgroups of PMCs who may be “at risk” for cognitive decline according to cognitive, behavioral, linguistic features?
3. To what extent does genetic variability (*FMR1* CGG repeat length and *APOE* alleles) predict cognitive-behavioral features among PMCs?

## 2 Materials and methods

### 2.1 Participants and procedures

#### 2.1.1 Participants: Arizona

Participants from the Arizona site included 47 *FMR1* PMCs (31F, 16 M) and 45 healthy controls (24F, 21 M) between 30 and 65 years of age (PMC *M* = 48.23 years, *SD* = 9.45; Control *M* = 43.95 years, *SD* = 9.24). All participants reported English as their primary language. Most participants reported their race as White (PMC: 93.75%, Controls: 62.22%) and had completed at least some college (>95%). Prior to participation, all PMCs provided documentation confirming *FMR1* premutation status. Follow-up genetic testing was completed on a subset of participants as part of a secondary genetic data collection protocol (see Genetics Collection and Analysis below). Inclusion criteria for PMCs required confirmed *FMR1* premutation status and the absence of significant neurological or developmental conditions directly affecting cognition, communication, or motor functioning that could not reasonably be attributed to premutation status (e.g., traumatic brain injury, stroke, or aphasia). Inclusion criteria for healthy controls were no known history of *FMR1*-associated conditions among immediate or extended family members, no history of neurological impairment, chronic medical conditions affecting cognition or motor functioning, or diagnosis of neurogenic speech, language, or cognitive disorders. Demographic characteristics are presented in Table 1.

**Table 1:** Participant demographics.

Demographic variables	PMCs *n* = 47	Healthy controls *n* = 45	Clinical MCI *n* = 12	Unimp. declining *n* = 71	Unimp. stable *n* = 290
Age, *M* (SD)	47.9 (9.3)	44.6 (9.5)	64.0 (6.6)	61.6 (6.24)	61.3 (6.6)
Sex	31F, 16 M	24F, 21 M	3F, 9 M	51F, 20 M	213F, 77 M
Education	—	—	—	—	—
College or higher	95.7% (45/47)	100% (45/45)	50.0% (6/12)	46.5% (33/71)	57.6% (167/290)
High School	4.3% (2/47)	0%	41.7% (5/12)	52.1% (37/71)	42.1% (122/290)
Less than HS	0%	0%	8.3% (1/12)	0%	0.3% (1/290)
Race	—	—	—	—	—
White	93.6% (44/47)	62.2% (28/45)	75.0% (9/12)	66.2% (47/71)	84.5% (245/290)
Black	0% (0/47)	15.6% (7/45)	25.0% (3/12)	31.0% (22/71)	12.4% (36/290)
More than one race	0% (0/47)	13.3% (6/45)	0%	1.4% (1/71)	0.3% (1/290)
Unknown/not reported	0% (0/47)	4.4% (2/45)	0%	1.4% (1/71)	2.8% (8/290)
Other	6.4% (3/47)	4.4% (2/45)	0%	0%	0%

PMC, premutation carrier; MCI, mild cognitive impairment; Unimp., unimpaired. PMCs and Healthy Controls were recruited at the University of Arizona (race coded per AZ-specific REDCap instrument: White, Black, more than one race, Other [American Indian/AN, Asian, Native Hawaiian/PI, Unknown, Prefer not to answer combined]). Clinical MCI, Unimpaired-Declining, and Unimpaired-Stable were recruited at the University of Wisconsin (race coded per separate WI demographic instrument: White, Black, More than one race, Unknown/not reported, Other [American Indian/AN, Asian, Native Hawaiian/PI, Other combined]).

Participants were recruited through the National Fragile X Foundation, the International Premutation Registry, Research Match, and community-based recruitment efforts. The study was approved by the University of Wisconsin and University of Arizona Institutional Review Boards, and all participants provided informed consent prior to enrollment.

#### 2.1.2 Participants: Wisconsin

Clinical comparison participants were drawn from the Wisconsin Registry for Alzheimer’s Prevention (WRAP), an ongoing longitudinal study designed to identify early risk and protective factors associated with ADRDs (Johnson et al., 2018). WRAP includes a large cohort of middle-aged and older adults (*n* ~ 1800) enriched for parental history of ADRDs, who have undergone repeated cognitive, behavioral, biomarker, and neuroimaging assessments since 2001.

Participants included in the present study (*n* = 373) were selected from the larger WRAP cohort. Participant selection criteria included: no diagnosis of dementia upon enrollment, but who either met criteria for MCI (*n* = 12), were unimpaired-declining (*n* = 71), and who were unimpaired-stable (*n* = 290) based on their most recent visit. All data included in the present study was based on the participant’s first time point. Classification procedures used to identify cognitively unimpaired and mild cognitive impairment (MCI) participants were based on previously reported methods (Langhough Koscik et al., 2021). Briefly, cognitive status was determined for each visit using a two-tiered consensus review process that incorporated both internal and published normative data. A multidisciplinary panel, including physicians, clinical neuropsychologists, and clinical nurse practitioners, first reviewed cases to determine whether MCI or dementia was present, and subsequently whether subclinical (cognitively unimpaired-declining; Langhough Koscik et al., 2021) status was present. The consensus team was blind to biomarker results. Diagnostic classifications were made according to NACC UDS criteria for MCI and dementia (National Alzheimer’s Coordinating Center, 2015; Petersen et al., 2021; Jack et al., 2024). The MCI label required that cognitive performance be lower than expected based on all available information, including prior performance, indicators of crystallized knowledge, and occupational and social history, and that this decline reach a threshold consistent with impairment, while not yet meeting criteria for dementia (McKhann et al., 2011; Langhough Koscik et al., 2021).

The Wisconsin cohort was included to provide clinically characterized comparison groups representing known trajectories of cognitive aging and decline. Because study site and group membership were completely confounded, site could not be included as a statistical covariate and cross-site comparisons are interpreted descriptively and with caution.

### 2.2 Characterization of cognition, behavior, and language

All participants in the Arizona cohort completed remote assessment of cognition, language, and behavior, including self-report questionnaires via Microsoft Teams. Data was collected and stored in REDCap (Harris et al., 2009, 2019). All sessions took approximately 2 h.

#### 2.2.1 Telephone Montreal cognitive assessment (T-MoCA)

In the Arizona sample, a screening for global cognitive functioning was assessed using the Telephone Montreal Cognitive Assessment (T-MoCA), a remote adaptation of the Montreal Cognitive Assessment designed for administration without visual stimuli (Katz et al., 2021). The T-MoCA has strong reliability with the in-person version of the MoCA(Katz et al., 2021). Total scores range from 0 to 22, with higher scores indicating better cognitive functioning. For exploratory subgroup analyses, PMCs were classified according to T-MoCA performance into PMC-Below (T-MoCA ≤ 17) and PMC-Above (T-MoCA ≥ 18) groups. This cutoff was selected based on prior work suggesting 81% specificity for mild cognitive impairment (Chappelle et al., 2023), and was therefore included as theoretically-defined subgroup for exploratory analyses based on cognitive screening.

#### 2.2.2 Language sample elicitation

Connected language samples were elicited using the Cookie Theft picture from the Boston Diagnostic Aphasia Examination (BDAE-3 Goodglass et al., 2001). Participants were instructed to describe everything happening in the picture using complete sentences. If a participant’s response was unusually brief, examiners provided the standardized prompt, “Do you see anything else going on?” This prompt was administered only once. Language samples from the Arizona cohort were an average of 61.59 s, and samples from the Wisconsin cohort were 50.4 s (SD = 0.02). At the Arizona site, audio and video were recorded via Microsoft Teams. For the Wisconsin site, samples were recorded using either an Olympus handheld recorder or an iPad 7-11th generation, placed flat on the table approximately 6 inches in front of the participant.

#### 2.2.3 Language sample processing and coding

Language samples were transcribed and coded in CHAT and CLAN software using standardized procedures (MacWhinney and Wagner, 2010). All files from the Arizona site were transcribed using the same procedures and guidelines established by the Wisconsin site. Transcribers were blind to participant diagnostic and cognitive status. Utterances were segmented into C-Units, indicating an independent clause and its modifiers. All transcribers were trained to 80% reliability with gold-standard files from TalkBank Dementia Bank on three files in a row. All files were transcribed by an initial coder, and a second transcriber reviewed for accuracy. Separate transcribers completed reliability for 20% of files, with accuracy calculated using the RELY program in CLAN software; average reliability for utterances was 92.99% (Range:80–100%) and average reliability for words was 94.96% (Range: 87–98%). All participants from the Arizona cohort were included in language sample analyses, and 371 participants from the Wisconsin cohort had available transcripts. For additional information on Wisconsin transcription and coding procedures, see (Mueller et al., 2018a).

Variables derived from the language samples include grammatical complexity, words per minute, unique words, type-token ratio, verbs per utterance, open/closed class ratio, and mean length of utterance in morphemes. See Table 2 for definitions.

**Table 2:** Variables derived from language sample analysis.

Language variable	Definition
Grammatical complexity	Number of grammatical relations that mark syntactic embeddings out of the total number of grammatical relations
Words per minute	Total number of words produced divided by sample duration
Unique words	Number of different words produced across the sample
Type token ratio	Number of unique words out of total words produced
Open/closed class ratio	Number of open-class words (nouns, verbs, adjectives, adverbs) by the number of closed-class words (pronouns, prepositions, conjunctions, determiners, auxiliaries).
Verbs per utterance	Average number of verbs used per utterance
Mean length of utterance (morphemes)	Average number of morphemes used per utterance

#### 2.2.4 Digit span

Short-term memory, auditory attention, and working memory were assessed using forward and backward digit span tasks derived from the Universal Data Set (UDS), Form C2, version 3.0 (National Alzheimer’s Coordinating Center, 2015). In the forward digit span task, participants repeated increasingly longer sequences of digits (up to nine) in the same order. Two trials were administered at each span length, and testing was discontinued following two consecutive incorrect responses at the same span. In the backward digit span task, participants repeated digit sequences in reverse order, up to eight digits. The maximum possible score for each task was 14. Forward digit span was interpreted as a measure of short-term memory, whereas backward digit span was interpreted as a measure of working memory.

#### 2.2.5 Semantic fluency

Semantic fluency was assessed using category fluency tasks, as indicated from the UDS (National Alzheimer’s Coordinating Center, 2015). Participants generated as many words as possible belonging to a specified semantic category within 60 s. Categories included animals and vegetables for Arizona, and only animals for Wisconsin; as such, only animals were included in analyses for cross-site comparisons. The primary outcome variable was the total number of correct responses. Repetitions, intrusions, and rule violations were excluded from scoring according to established procedures (Mueller et al., 2015).

#### 2.2.6 Phonemic fluency

Phonemic fluency was assessed using letter fluency tasks from the UDS (National Alzheimer’s Coordinating Center, 2015). Participants generated as many words as possible beginning with a specified letter within 60 s. Letters included F, L, and B for Arizona, and C, F, and L for Wisconsin. The primary outcome variable was the total number of correctly produced words. Repetitions, proper nouns, and rule violations were excluded from scoring (Mueller et al., 2015).

#### 2.2.7 Center for epidemiologic studies depression scale (CES-D)

Depression symptoms were assessed using the Center for Epidemiologic Studies Depression Scale (CES-D; Radloff, 1977). The CES-D is a 20-item self-report measure assessing depression symptoms experienced during the previous week. Items are rated on a four-point scale ranging from 0 to 3, yielding total scores from 0 to 60. Higher scores indicate greater depression symptom severity.

### 2.3 Genetics collection and analysis

A subset of Arizona participants participated in a secondary genetic data collection protocol. Of those contacted, 26 PMCs (18 females, 8 males) and 24 healthy controls (14 females, 10 males) completed genetic sampling. Buccal samples were collected using Asuragen collection kits distributed and returned by mail. Genetic data completion did not differ significantly by age, education, sex, group membership (PM vs. Control), short-term memory, working memory, depression symptoms, or semantic verbal fluency (all *p*s > 0.34). One isolated difference emerged on phonemic fluency (*p* = 0.028), consistent with the expected false-positive rate across multiple comparisons and not indicative of a systematic missingness pattern. These results suggest that genetic data are missing completely at random within the AZ subsample.

#### 2.3.1 *FMR1* analysis

Total DNA was isolated from a buccal swab (Whatman OmniSwab) in 750 μL lysis buffer (50 mM Tris pH 8.0; 50 mM EDTA; 25 mM Sucrose; 100 mM NaCl; 1% SDS) by overnight lysis with proteinase K at 55 °C (Invitrogen/ThermoFisher), followed by robotic DNA extraction using a Kingfisher FLEX robotic magnetic-particle purification system (ThermoFisher) and MagMAX™ DNA Multi-Sample Ultra 2.0 Kit bead extraction chemistry (Applied Biosystems/ThermoFisher).

We quantified recovered DNA using a BioTEK Synergy HT plate reader (BioTEK/Agilent) and QuantiFluor dsDNA reagent (Promega), then diluted stock DNA to 5 ng/μl in 1X Low TE (10mMTris-pH 8.0, 0.1 mM EDTA). DNA was used at 10–20 ng per reaction in the AmplideX PCR/CE FMR1 kit (Asuragen) and PCR products were resolved by capillary electrophoresis on a DNA Analyzer 3,730 (Applied Biosystems/ThermoFisher). CGG repeats and mutation analysis was performed using AmplideX PCR/CE Reporter Software (Asuragen) per manufacturer’s instructions.

#### 2.3.2 *APOE* analysis

For the Arizona data, two sets of Taqman reactions were performed, an initial 384-well format plate with everything being reacted, and a second 96-well format plate for necessary re-reactions. Both plates were reacted using the ABI Taqman Fast Advanced Master Mix and ABI Taqman Made-to-Order Genotyping Assays, per the manufacturer’s recommendations. We set up the genotyping reactions in 15uL volumes. The 384-well plate was reacted on our ABI 7900HT qPCR instrument, and the 96-well plate was reacted on our ABI 7300 qPCR instrument. The reaction protocol is the same on both instruments, the only difference is in the plate format, and the results can be considered interchangeable between either platform. In both reaction plates, the Beckman Biomek FX robot was used to transfer the DNA samples into the qPCR plate for reaction. 10 ng of genomic DNA was used per reaction. For these Taqman genotyping reactions, the genomic DNA was dried down in the well in a vacuum desiccation chamber, and the Taqman genotyping assay mixture was loaded into the plate a day later. After the genotyping plates were reacted and scanned in the qPCR instrument, the results were exported to Excel. The re-reacted DNA samples were updated, correcting the failed initial reactions from the 384-well plate. The Taqman genotyping reaction results (VIC/FAM) for both SNPs were translated into nucleotides (CC/CT/TT). These nucleotide results were then used to translate to *APOE* Genotype categories (E2/E3/E4). For analytic purposes, *APOE* genotype was collapsed into ε4 carrier versus non-carrier status. Details about WRAP genomic data collection and quality control have been described previously (Darst et al., 2019).

### 2.4 Analysis plan

All analyses were conducted in R (version 4.6). Prior to primary analyses, demographic equivalence across groups was assessed using one-way ANOVAs and chi-square tests. Participant sex, age, and education showed significant group differences and were included as covariates throughout. A significant correlation between semantic and phonemic verbal fluency (*r* = 0.37, *p* < 0.001) warranted inclusion of phonemic fluency as an additional covariate in all semantic fluency models. Because group membership was perfectly collinear with site, site could not be included was a covariate and cross-site comparisons should be interpreted with caution given some demographic differences between the samples, particularly in age (AZ *M* = 46.3 years; WI *M* = 61.4 years).

For Aim 1, PMCs were compared descriptively to healthy controls and to the three WI clinical reference groups (Clinical MCI, Unimpaired-Declining, Unimpaired-Stable) across measures of short-term memory, working memory, depression symptoms, and semantic verbal fluency, in order to evaluate the extent to which PMCs show cognitive-behavioral similarities to individuals with pre/MCI. One-way ANOVAs were conducted for each outcome with group as the between-subjects factor; FDR correction was applied across the four primary cognitive-affective outcomes, and both corrected and uncorrected *p*-values are reported. Pairwise effect sizes (Cohen’s *d*) were computed for all group comparisons. Given the demographic confound between site and group, cross-site comparisons involving the WI reference groups are interpreted descriptively rather than as definitive evidence of phenotypic overlap or divergence.

For Aim 2, PMCs were classified into subgroups based on T-MoCA performance (PMC-Below, ≤ 17; PMC-Above, ≥18) and compared across measures of short-term memory, working memory, depression symptoms, semantic verbal fluency, and connected speech. Given small subgroup sample sizes, Wilcoxon rank-sum tests were used for all subgroup comparisons, with Cohen’s *d* reported alongside statistical significance to characterize effect magnitude independent of sample size. To contextualize PM subgroup differences within the broader sample, one-way ANOVAs were conducted for each outcome with group as the between-subjects factor. Given the exploratory nature of PMC subgroup comparisons and the small sample size, *p*-values for Aim 2 analyses are reported without correction for multiple comparisons. Findings should be interpreted accordingly, with emphasis placed on effect sizes and confidence intervals as indicators of practical significance.

For Aim 3, CGG repeat length data from two assay sources were combined after confirming near-perfect agreement (*r* = 0.988), yielding data for 46 of 47 PMCs. CGG repeat length was mean-centered with linear and quadratic terms included in all models. *APOE* genotype was collapsed to ε4 carrier versus non-carrier. *APOE* genotype data were available for 26 PMC and 24 Control participants from the AZ sample. Among PMCs, 18 (75%) were ε3/ε3 homozygous, 5 (20.8%) carried the ε4 allele, and 2 (8.3%) carried the ε2 allele. Among healthy controls, 12 were ε3/ε3 homozygous, 11 (44%) carried the ε4 allele, and 2 (8%) carried the ε2 allele. Multiple regression models were fit for each outcome, including CGG linear and quadratic terms, *APOE* ε4 status, a CGG × *APOE* interaction, and covariates. The CGG × *APOE* interaction is treated as exploratory given limited power. All Aim 3 findings are considered preliminary and hypothesis-generating.

## 3 Results

### 3.1 Descriptive statistics

The full sample comprised 465 participants across five groups (see Table 1). Groups differed significantly on age, education, and sex, reflecting the multi-site design and the expected demographic differences between the younger AZ premutation sample and the older WI clinical comparison groups.

### 3.2 Aim 1. Cross-site group comparisons

As described above, cross-site comparisons should be interpreted descriptively given confounding across sites. Nevertheless, statistical comparisons were evaluated to glean preliminary information regarding the influence of group status on cognitive-linguistic performance.

Cognitive, affective, and language characteristics by group are presented in Table 3. Relative to healthy controls, PMCs showed a significant group-level elevation in depression symptoms after covarying age, sex, and education (*d* = 0.55; see Tables 4, 5). No significant within-site differences were observed on memory, verbal fluency, or T-MoCA total score. To address whether PMCs showed cognitive-behavioral similarities to individuals with pre/MCI, we descriptively compared PMCs to the three WI clinical reference groups (Clinical MCI, Unimpaired-Declining, Unimpaired-Stable) using one-way ANOVAs across all four primary outcomes (see Table 5) and connected speech variables from the Cookie Theft picture description task; cross-site comparisons are interpreted descriptively given substantial demographic differences between sites. PMCs showed medium-to-large effect sizes relative to all three WI groups, with higher scores on digit span and semantic verbal fluency (*d*s ranging from 0.38 to 1.40), and a small-to-medium effect reflecting higher depression symptoms relative to Unimpaired-Stable (*d* = 0.81). PMCs did not differ significantly from any group on words per minute (*F*(4, 453) = 1.89, *p* = 0.111), unique word count (*F*(4, 456) = 1.67, *p* = 0.157), open/closed class ratio (*F*(4, 456) = 0.46, *p* = 0.762), propositional density (*F*(4, 456) = 1.76, *p* = 0.136), or noun/verb ratio (*F*(4, 456) = 0.80, *p* = 0.526). However, PMCs showed significantly lower utterance length (mean length of utterance in morphemes) relative to the WI Unimpaired-Declining (*t*(456) = 3.20, *p* = 0.002) and Unimpaired-Stable groups (*t*(456) = 4.72, *p* < 0.001), and fewer verbs per utterance relative to Unimpaired-Declining (*t*(456) = 2.06, *p* = 0.040) and Unimpaired-Stable (*t*(456) = 3.64, *p* < 0.001), a pattern consistent with the reduced syntactic complexity observed within the PM-Below subgroup (see Aim 2 below).

**Table 3:** Cognitive, affective, and language characteristics by group.

Cognitive-behavioral variables	PMC *n* = 47	Control *n* = 45	Clinical MCI *n* = 12	Unimp. declining *n* = 71	Unimp. stable *n* = 290
Cognitive and affective measures					
Digit span forward	8.83 (2.41)	8.87 (2.41)	10.7 (1.79)	9.72 (2.17)	10.3 (2.19)
Digit span backward	7.89 (2.06)	8.29 (2.36)	6.00 (1.34)	6.62 (2.35)	7.20 (2.05)
Depression symptoms	12.3 (10.3)	7.56 (6.60)	8.91 (11.2)	8.97 (9.06)	6.48 (6.53)
SVF animals	27.0 (7.43)	27.9 (5.26)	21.0 (4.35)	20.8 (5.00)	22.1 (5.10)
Phonemic fluency	46.3 (9.81)	49.1 (14.7)	37.4 (10.1)	38.8 (10.2)	44.5 (11.1)
T-MoCA^a^	19.0 (2.18)	19.8 (1.71)	—	—	—
T-MoCA ≤17, *n* (%)^a^	14 (29.8%)	0 (0%)	—	—	—
Language (cookie theft)					
Words per minute	135 (23.9)	133 (29.7)	120 (28.0)	115 (35.8)	127 (31.8)
Unique words	68.7 (29.6)	68.5 (27.8)	54.2 (23.5)	51.9 (18.2)	56.6 (21.0)
Type-token ratio	0.56 (0.10)	0.54 (0.10)	0.60 (0.12)	0.61 (0.09)	0.62 (0.09)
Open/closed ratio	0.97 (0.15)	0.92 (0.14)	1.00 (0.25)	0.98 (0.20)	0.99 (0.28)
Mean length of utterance (morphemes)	9.74 (2.93)	10.5 (2.44)	9.54 (1.80)	10.7 (2.82)	11.4 (3.49)
Verbs per utterance	1.42 (0.51)	1.44 (0.38)	1.33 (0.35)	1.45 (0.47)	1.58 (0.51)
Grammatical Complexity^a^	0.09 (0.03)	0.10 (0.02)	–	–	–

Values are *M* (SD) unless otherwise noted. PMC, premutation carriers; MCI, mild cognitive impairment.

^a^T-MoCA and Grammatical Complexity data available for AZ site only (PM: *n* = 46, Control: *n* = 45).

**Table 4:** One-way ANCOVA results for primary outcome variables across groups.

Outcome/predictor	df	*F*	*p*	*p* (FDR)	η^2^_p_
Digit span forward (short-term memory)					
**Group**	**4**	**7.04**	**<0.001**	**<0.001**	**0.07**
Age	1	0.57	0.451	—	<0.01
Sex	1	0.75	0.388	—	<0.01
**Education**	**1**	**14.90**	**<0.001**	**—**	**0.03**
Digit span backward (working memory)					
**Group**	**4**	**2.57**	**0.037**	**0.037**	**0.05**
Age	1	1.21	0.273	—	<0.01
Sex	1	0.31	0.577	—	<0.01
**Education**	**1**	**16.63**	**<0.001**	**—**	**0.04**
CES-D (depression symptoms)					
**Group**	**4**	**5.68**	**<0.001**	**<0.001**	**0.06**
**Age**	**1**	**4.59**	**0.033**	**—**	**<0.01**
**Sex**	**1**	**6.75**	**0.010**	**—**	**0.01**
**Education**	**1**	**3.94**	**0.048**	**—**	**<0.01**
SVF animals (semantic verbal fluency)					
**Group**	**4**	**4.33**	**0.002**	**0.003**	**0.04**
Age	1	3.69	0.055	—	0.02
Sex	1	0.10	0.749	—	<0.01
Education	1	3.61	0.058	—	0.03
**Phonemic fluency**	**1**	**38.27**	**<0.001**	**—**	**0.08**

Type III sums of squares. Covariates: age, sex, education (+ phonemic fluency for SVF). df error: DSF/DSB = 455, CES-D = 453, SVF = 442. FDR correction applied across the four group-level tests only. Bold *p*-values indicate significance at *α* = 0.05. All five groups included in omnibus ANCOVA: PM carriers (*n* = 47), healthy controls (*n* = 45), Clinical MCI (*n* = 12), Unimpaired-Declining (*n* = 71), and Unimpaired-Stable (*n* = 290).

**Table 5:** Pairwise Cohen’s *d* effect sizes for PMCs versus comparison groups.

Comparison	DSF		DSB		CES-D		SVF	
	*d*	95% CI	*d*	95% CI	*d*	95% CI	*d*	95% CI
PMC vs. Control^a^	−0.02	[−0.42, 0.39]	−0.18	[−0.59, 0.23]	**0.55**	**[0.13, 0.96]**	−0.14	[−0.55, 0.27]
PMC vs. Clinical MCI^b^	**−0.82**	**[−1.50, −0.15]**	**0.97**	**[0.29, 1.65]**	0.33	[−0.33, 0.98]	**0.86**	**[0.21, 1.52]**
PMC vs. Unimp. declining^b^	**−0.39**	**[−0.76, −0.02]**	**0.57**	**[0.19, 0.95]**	0.35	[−0.02, 0.72]	**1.02**	**[0.63, 1.41]**
PMC vs. Unimp. Stable^b^	**−0.67**	**[−0.98, −0.36]**	**0.34**	**[0.03, 0.65]**	**0.81**	**[0.50, 1.13]**	**0.89**	**[0.58, 1.21]**

Positive values indicate PMCs (premutation carriers) scored higher than the comparison group. 95% CIs in brackets. Within-site comparisons (PMC vs. Control) are shaded. Bold values indicate 95% CI excludes zero. Effect size benchmarks: small = 0.20, medium = 0.50, large = 0.80 (Cohen, 1988). DSF, Digit span forward task (short-term memory); DSB, Digit span backward task (working memory); CES-D, Depression symptoms; SVF, Semantic verbal fluency.

^a^Within-site comparison (both AZ). Shaded row.

^b^Cross-site comparison (PMC = AZ; MCI groups = WI). Differences may partly reflect site- or administration-related variance; interpret with caution.

A separate within-site analysis restricted to the AZ sample (PMCs vs. healthy controls only) found no significant language differences between these two groups specifically; education was the dominant predictor across language variables in this within-site model (Table 5).

### 3.3 Aim 2: group comparisons and PM subgroup characterization

**Figure 1:**
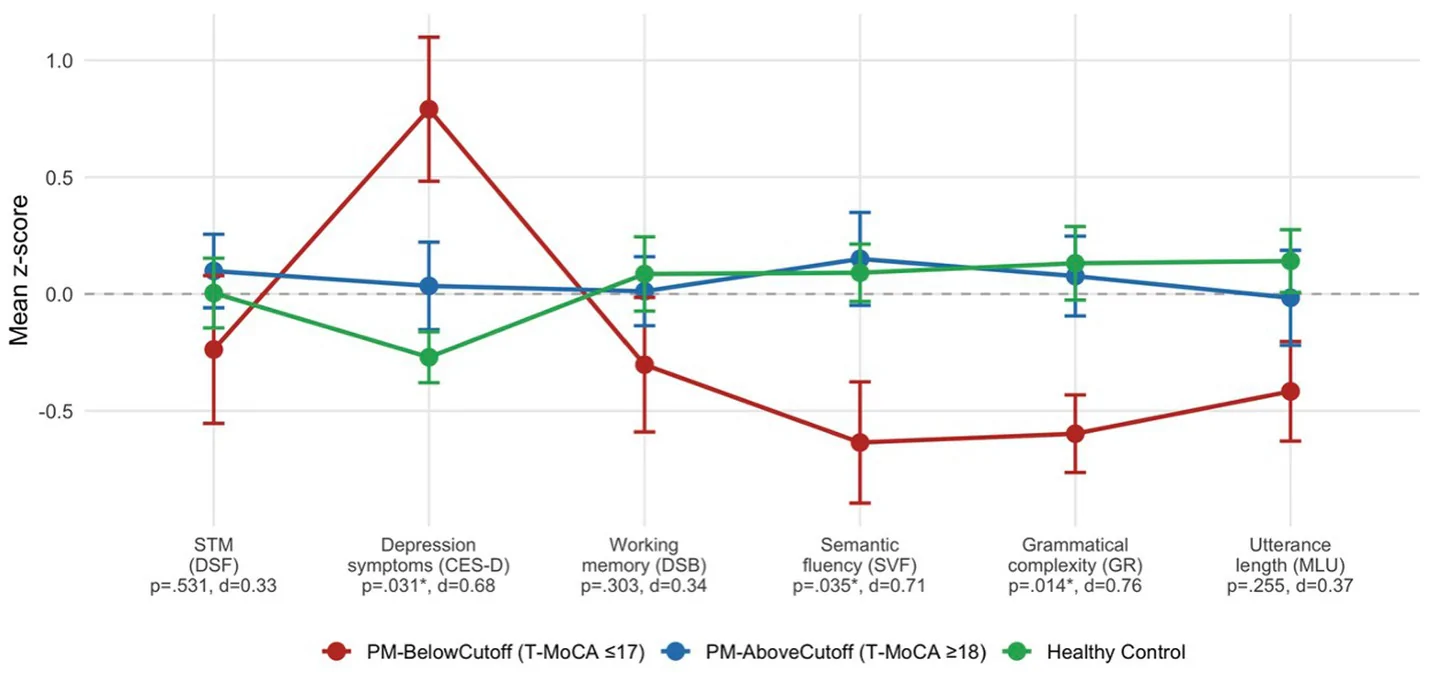
Cognitive, mood, and linguistic profile of *FMR1* premutation carrier subgroups defined by T-MoCA performance, with healthy controls as reference. Mean *z*-scores (±SE) are shown for PMC-Below Cutoff (*n* = 14, T-MoCA ≤17), PM-Above Cutoff (*n* = 32, T-MoCA ≥18), and healthy controls (*n* = 45). Z-scores were computed relative to the full AZ analytic sample. The dashed line represents the sample mean. STM, short-term memory (Digit Span Forward); CES-D, Center for Epidemiologic Studies Depression Scale; DSB, Digit Span Backward; SVF, semantic verbal fluency (animals); GR, grammatical complexity index (proportion of complex grammatical relations); MLU, mean length of utterance in morphemes. *p*-and *d*-values shown below each measures reflect PMC-Below vs. PM-Above Cutoff Wilcoxon rank-sum comparisons. *p < 0.05.

All Aim 2 findings are considered preliminary and hypothesis-generating pending replication in larger samples. The two PMC subgroups were demographically comparable but diverged meaningfully across cognitive, affective, and linguistic measures (see Table 6 and Figure 1). Within the PMC group, 14 participants (29.8%) scored at or below the T-MoCA clinical cutoff, constituting the PMC-Below subgroup. The remaining 32 (68.1%) scored above the cutoff.

**Table 6:** Characteristics of PMC subgroups defined by T-MoCA cutoff score.

Variables	PMC-Below *n* = 14	PMC-Above *n* = 32	Control *n* = 45	*p* ^a^	*d*[95% CI]
Cognitive and affective measures					
Digit span forward	8.29 (2.95)	9.09 (2.18)	8.87 (2.41)	0.531	0.33 [−0.96, 0.30]
Digit span backward	7.43 (2.47)	8.12 (1.88)	8.29 (2.36)	0.303	0.34 [−0.97, 0.29]
Depression symptoms	17.1 (10.8)	10.3 (9.72)	7.56 (6.60)	0.031*	0.68 [0.04, 1.32]
SVF animals	23.3 (6.43)	28.3 (7.31)	27.9 (5.26)	0.035*	0.71 [0.06, 1.36]
Phonemic fluency	46.7 (9.99)	46.0 (10.0)	49.1 (14.7)	0.818	0.07 [−0.56, 0.70]
Language (Cookie theft)					
Words per minute	130 (26.2)	138 (23.1)	132 (29.7)	0.349	0.33 [−0.95, 0.31]
Unique words	62.6 (28.2)	72.0 (30.6)	68.5 (27.8)	0.271	0.32 [−0.31, 0.32]
Type-token ratio	0.58 (0.10)	0.55 (0.10)	0.54 (0.10)	0.346	0.25 [−0.38, 0.88]
Open/closed ratio	0.94 (0.13)	0.99 (0.15)	0.92 (0.14)	0.162	0.34 [−0.99, 0.29]
Utterance length (MLU morphemes)	8.98 (2.24)	10.1 (3.22)	10.5 (2.44)	0.192	0.37 [–1.01, 0.27]
Verbs per utterance	1.26 (0.28)	1.47 (0.58)	1.44 (0.38)	0.110	0.42 [−1.06, 0.22]
Grammatical complexity^b^	0.08 (0.02)	0.10 (0.03)	0.10 (0.03)	0.014*	0.76 [−1.41, −0.11]

Values are *M* (SD) unless otherwise noted. Healthy controls included as reference. PMC-Below = PMC Below Cutoff (T-MoCA ≤17); PMC-Above = PMC Above Cutoff (T-MoCA ≥18). *p*-and *d*-reflect PMC-Below vs. PMC-Above comparisons only. *p < 0.05.

^a^Wilcoxon rank-sum tests used for all comparisons given small subgroup sample sizes.

^b^Grammatical complexity = proportion of complex grammatical relations out of all grammatical relations.

The PMC-Below group showed significantly higher depression symptoms, lower semantic verbal fluency, and reduced grammatical complexity relative to the PMC-Above group (all Wilcoxon *p*s < 0.04, all *d*s > 0.68; see Table 6 for exact statistics). Beyond these significant findings, a consistent pattern of small-to-medium effects in the expected direction across additional cognitive and linguistic measures, including mean length of utterance in morphemes (*d* = 0.37), verbs per utterance (*d* = 0.42), words per minute (*d* = 0.33), and unique word count (*d* = 0.32), suggests a broader pattern of reduced linguistic complexity and output that likely reflects limited statistical power given the small PMC-Below sample size (*n* = 14) rather than a true absence of effect. The PMC-Above group was largely indistinguishable from healthy controls across all phenotypic measures.

**Figure 2:**
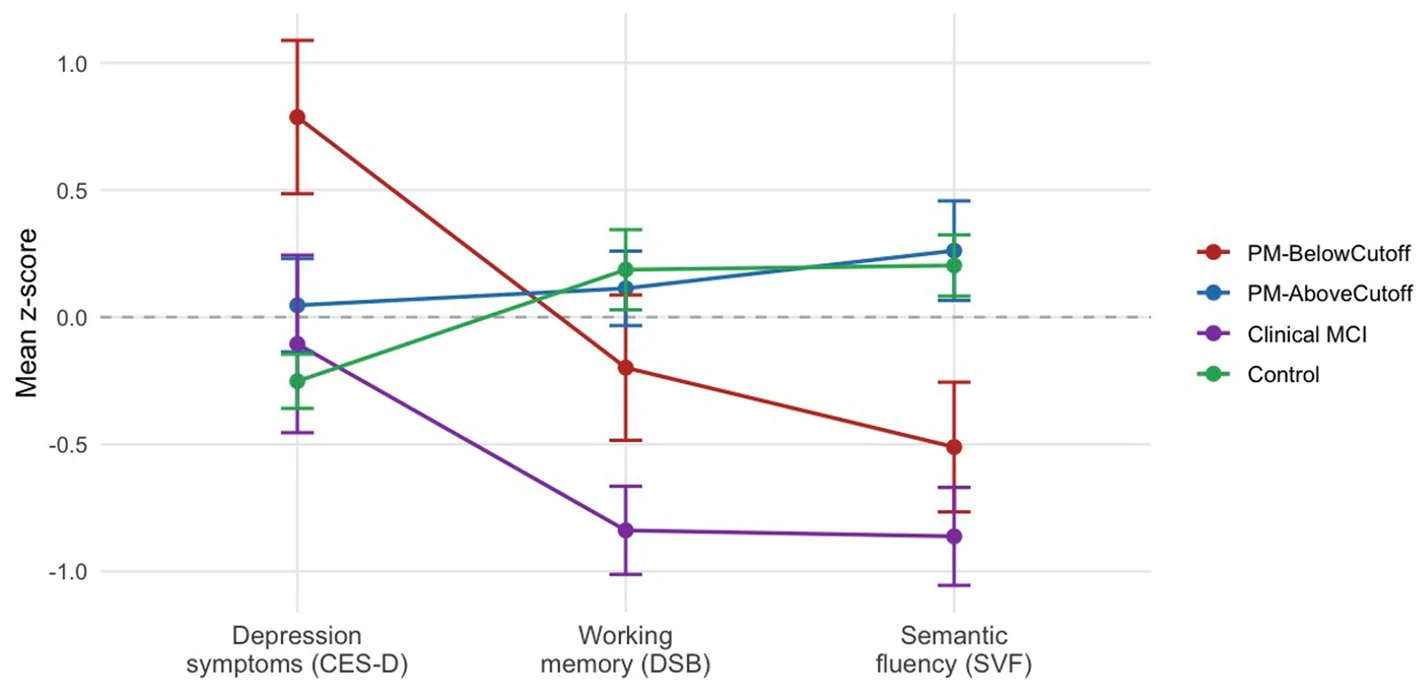
Depression, cognition, and language performance across PM carrier subgroups and clinical reference groups. Mean *z*-scores (±SE) are shown for PMC-Below Cutoff (*n* = 14, T-MoCA ≤17), PM-Above Cutoff (*n* = 32, T-MoCA ≥18), Clinical MCI (*n* = 12), and healthy controls (*n* = 45). *Z*-scores were computed relative to the full analytic sample. The dashed line represents the sample mean. CES-D, Center for Epidemiologic Studies Depression Scale; DSB, Digit Span Backward; SVF, semantic verbal fluency (animals). PM subgroups and healthy controls were recruited at the University of Arizona (AZ); Clinical MCI was recruited at the University of Wisconsin (WI). Cross-site comparisons should be interpreted with caution given substantial demographic differences between sites, particularly in age.

Exploratory analyses examined cross-site comparisons with the WI clinical reference sample. PMC-Below carriers showed a qualitatively different pattern from the Clinical MCI reference group (see Figure 2), characterized by elevated depression and reduced grammatical complexity. This pattern diverged similarly from the Unimpaired-Declining and Unimpaired-Stable WI reference groups, both of which showed lower depression symptoms (*M* = 8.97 and 6.48, respectively) and higher semantic verbal fluency (*M* = 20.8 and 22.1, respectively) than PMC-Below carriers (*M* = 17.1 and 23.3; see Table 2), though these cross-site comparisons should be interpreted with the same caution noted above given demographic differences between sites. Across all three WI reference groups, none showed the combination of elevated depression alongside relatively preserved working memory that characterized PMC-Below carriers, reinforcing that this profile is distinct from the broader spectrum of cognitive impairment represented in the WI sample rather than specific to one comparison group. Exploratory analyses examined intercorrelations among the three significant subgroup outcomes within PMCs. CES-D, SVF, and grammatical complexity were largely independent of one another (all *r*s < 0.25, all *p*s > 0.10), suggesting distinct rather than overlapping dimensions of a possible “at-risk” profile. Sex did not moderate any subgroup finding (all interaction *p*s > 0.32), indicating that the observed differences were consistent across male and female PMCs.

### 3.4 Aim 3: genetic predictors of cognitive-behavioral outcomes

To examine whether CGG repeat length predicted cognitive-behavioral outcomes within PMCs specifically, multiple regression models were fit in the PM-only subsample (*n* = 46 with available CGG data; see Supplementary Table 1 for full regression coefficients and confidence intervals). CGG repeat length did not significantly predict depression symptoms (*p* = 0.124), short-term memory (*p* = 0.769), working memory (*p* = 0.273), or semantic verbal fluency (*p* = 0.579). The quadratic CGG term was non-significant across all outcomes (all *p*s > 0.72), indicating no evidence of a curvilinear relationship across the observed premutation range (56–135 repeats). *APOE* carrier status (any variant vs. ε3/ε3 reference) did not significantly predict any outcome (depression symptoms: *p* = 0.347; short-term memory: *p* = 0.299; working memory: *p* = 0.426; semantic verbal fluency: *p* = 0.742; see Supplementary Table 1 for regression statistics). The low ε4 carrier rate observed in PMCs (20.8%, *n* = 5) relative to healthy controls (44%, *n* = 11) is consistent with prior work suggesting *APOE* ε4 may be more relevant to advanced FXTAS than to non-FXTAS premutation status. The CGG × *APOE* interaction was non-significant across all outcomes and should be interpreted with caution given that only five PMCs carried the *APOE* ε4 allele. Notably, PMC subgroups did not differ on CGG repeat length (*t*(21.7) = 0.62, *p* = 0.543), and the CGG × T-MoCA subgroup interaction on depression symptoms was non-significant (*p* = 0.763), indicating that the subgroup difference in depression symptoms observed in Aim 2 is independent of repeat length. All Aim 3 findings are considered preliminary and hypothesis-generating pending replication in larger samples.

## 4 Discussion

This study aimed to compare cognitive, linguistic, and behavioral features *FMR1* PMCs, controls, and individuals with known progression to MCI. Given differences in study population characteristics, findings require cautious interpretation. Nevertheless, within-sample characteristics of the *FMR1* premutation suggest a notable profile distinct from individuals who progressed to MCI, suggesting potential differences in cognitive aging across populations.

The most consistent finding across analyses was the central role of depression symptoms in characterizing the potentially at-risk PMC subgroup. The PMC-Below subgroup, characterized by cognitive screening on the T-MoCA, exhibited elevated CES-D scores relative to the PMC-Above subgroup and healthy controls, independent of CGG repeat length, sex, and the other cognitive and linguistic features that distinguished the subgroups. The independence of depression symptoms from semantic verbal fluency and grammatical complexity, three largely uncorrelated dimensions of this subgroup group, suggests that affective dysregulation is not simply a downstream consequence of cognitive decline but may represent a distinct and potentially early manifestation of *FMR1*-related vulnerability. Longitudinal studies will be required in order to determine this possibility.

These findings are consistent with prior work documenting elevated rates of mood disorders in PMCs and extend the literature in two ways. First, within PMCs, those who meet clinical threshold for cognitive impairment on the T-MoCA show higher depression symptom burden than those who do not, suggesting that depression may index risk for more advanced neurocognitive involvement rather than reflecting a uniform feature of PMC status. This is consistent with findings from case studies of female PMCs prior to FXTAS onset who evidenced broad neuropsychiatric involvement (Liani et al., 2025). Longitudinal work tracking depression symptoms alongside cognitive and neurological markers across the PMC lifespan is needed to establish whether depression precedes, accompanies, or follows cognitive decline in this population to establish depression as a possible marker of FXTAS progression.

A secondary but theoretically important finding was the qualitative dissociation between PMC-Below and the Clinical MCI reference group. Whereas Clinical MCI was characterized by reduced working memory and relatively low depression symptoms, PMC-Below featured average working memory alongside markedly elevated depression and reduced grammatical complexity. This dissociation suggests that cognitive risk in PMCs does not simply recapitulate the amnestic phenotype characteristic of Alzheimer’s disease and argues against framing PMC-related impairment as a variant of MCI. Rather, this PMC subgroup may reflect a distinct neurodegenerative trajectory — one in which affective and linguistic systems are preferentially vulnerable at early stages, consistent with emerging evidence of frontotemporal and cerebellar involvement in FXTAS pathology (Cabal-Herrera et al., 2020). Notably, the majority of PMCs in this sample (68.1%) showed a cognitive-affective profile statistically indistinguishable from healthy controls, underscoring that cognitive-affective vulnerability is not a universal feature of PMC status and that the profile described here characterizes a meaningful but bounded subset of carriers.

One intriguing finding was the role of grammatical complexity in differentiating the PMC subgroups. In a longitudinal study, Klusek et al. (2022b) found that maternal PMCs with a family history of FXTAS evidenced stepper declines in grammatical complexity relative to those without a family history of FXTAS. Likewise, in a case study of a male with FXTAS, Grigsby et al. (2006) identified that complex grammar production was an area of difficulty. In contrast, Maltman et al. (2024) found that mean length of utterance (a common marker of grammatical complexity) was not a significant predictor of FXTAS symptom severity in a sample of males and females. Thus, the sensitivity of grammatical complexity to FXTAS-related risk may be dependent on either the context of the language sample (narrative vs. monologue) or how the grammatical complexity variable is constructed, as all studies used different definitions of complexity. Further work is needed to identify the sensitivity of linguistic markers of cognitive aging relevant to PMC-related conditions, as they have been shown as promising predictors of cognitive decline in MCI/ADRDs.

A further notable feature of the PMC-Below subgroup was the relative independence of its defining characteristics. Depression symptoms, semantic verbal fluency, and grammatical complexity were only weakly and non-significantly correlated with one another within PMCs, suggesting that these represent distinct rather than overlapping markers of risk. This has two implications. First, it suggests that no single measure is likely to fully capture PMC-related cognitive-affective vulnerability, and that a multidomain assessment approach spanning affective, semantic, and syntactic domains may be necessary to adequately characterize risk in this population. Second, the relative independence of these dimensions raises questions about whether they reflect a common underlying neurodegenerative process unfolding along different timescales, or genuinely distinct (if co-occurring) vulnerabilities with potentially different mechanistic origins. Distinguishing between these possibilities will require longitudinal designs capable of tracking the temporal ordering of symptom emergence across domains.

Genetic analyses did not reveal significant predictors of cognitive-behavioral outcomes within PMCs in this preliminary sample. CGG repeat length and *APOE* ε4 carrier status were not associated with depression symptoms, memory, or verbal fluency after covarying for age, sex, and education. CGG repeat length did not significantly predict depression symptoms within PMCs in this preliminary sample, though prior research suggests inconsistent links to CGGs in the premutation (Hessl et al., 2005; Roberts et al., 2009, 2016; Klusek et al., 2025). Larger samples will be needed to adequately test whether a dose–response relationship exists between repeat length and depression within the premutation range. Further, phenotypic variability in the sample was not explained by *APOE* genotype. *APOE* ε4 carrier status did not predict any cognitive-affective outcome, and PMCs in this sample showed a numerically lower ε4 carrier rate (20.8%) than healthy controls (44%). This null finding is itself informative, although it should be interpreted with caution and replicated in larger, more diverse samples, as only a subset of our larger sample completed genetic testing, and participants were largely in early-middle age. Prior work has shown higher rates of *APOE* ε4 expression in PMCs with FXTAS (Silva et al., 2013), but the present sample consisted of non-FXTAS PMCs, raising the possibility that *APOE*-related risk may be specific to more advanced stages of PMC-associated neurodegeneration rather than present from early cognitive-affective involvement. It is also possible that links to cognitive-behavioral variability may not be apparent until older age, as *APOE* effects on cognitive decline and neurodegeneration become more pronounced with age in the general population (Chen et al., 2025). To our knowledge, this is among the first studies to examine *APOE* genotype in a non-FXTAS PMC sample, and the absence of an effect here may help delineate the boundary conditions under which *APOE* contributes to PMC-related outcomes.

### 4.1 Limitations and future directions

This study represents a unique approach to improving our understanding of early markers of potential cognitive decline but was limited in a number of ways. First, the study is inherently limited by the cross-sectional design. Though we identified promising patterns that distinguished subgroups of PMCs, without a longitudinal design, the designation of PMCs as “at-risk” is tentative. Future studies should examine retrospective patterns relevant to cognition and depression to more effectively determine the utility of the profiles observed here. We were also limited in our capacity to match samples across studies. The separate clinical populations and similar study protocols enabled unique insight into cross-population comparisons, though the age differences alongside procedural variation (in-person vs. remote testing) precluded statistical comparison of group performance. Nevertheless, this study provides hypothesis-generating findings that will lay the groundwork for understanding symptom trajectories across conditions. Together, these findings highlight a constellation of phenotypic features relevant to disease tracking that may be integrated into clinical studies of PMCs in future work. The relative ease and low cost of administration for the T-MoCA, CES-D, and semantic verbal fluency tasks may prove useful clinical tools for future determination of relative risk, though larger sample sizes and longitudinal studies are needed to clarify clinical utility.

## Data Availability

The datasets analyzed for this study can be accessed upon reasonable request in accordance with data sharing policies from the University of Arizona and University of Wisconsin.
