# Supplementary material for: Cognitive-behavioral phenotypes in FMR1 premutation carriers: a cross-population comparison with mild cognitive impairment
Source: Front Mol Neurosci. 2026 Sep 10;19:1943587. doi: 10.3389/fnmol.2026.1943587 (PMC13602314; doi:10.3389/fnmol.2026.1943587)
Supplement: Supplementary file 1 [file Table_1.docx]

**Supplementary Table 1.** Regression coefficients for CGG repeat length and *APOE* predicting cognitive-behavioral outcomes (PM carriers only)

| Predictor | DSF | | DSB | | CES-D | | SVF | |
| --- | --- | --- | --- | --- | --- | --- | --- | --- |
|  | B | 95% CI | B | 95% CI | B | 95% CI | B | 95% CI |
| Primary predictors | | | | | | | | |
| CGG (linear) | 0.003 | [−0.027, 0.033] | −0.007 | [−0.030, 0.016] | 0.138 | [−0.051, 0.327] | 0.032 | [−0.063, 0.127] |
| CGG (quadratic) | <0.001 | [−0.001, 0.001] | <0.001 | [−0.001, 0.001] | 0.001 | [−0.005, 0.007] | 0.001 | [−0.002, 0.004] |
| *APOE* any variant | 0.191 | [−1.626, 2.008] | −0.208 | [−1.604, 1.188] | −0.681 | [−7.536, 6.174] | 1.391 | [−1.998, 4.780] |
| Covariates | | | | | | | | |
| Age | 0.057 | [−0.008, 0.122] | 0.064 | [0.014, 0.114] | −0.373 | [−0.618, −0.128] | 0.014 | [−0.107, 0.135] |
| Sex | 0.554 | [−0.749, 1.857] | 0.483 | [−0.521, 1.487] | −0.327 | [−5.247, 4.593] | 2.127 | [−0.313, 4.567] |
| Education | 0.216 | [−0.171, 0.603] | 0.168 | [−0.129, 0.465] | −1.134 | [−2.595, 0.327] | 0.540 | [−0.183, 1.263] |
| Model fit | | | | | | | | |
| R² | .143 | | .228 | | .222 | | .118 | |
| Adjusted R² | .023 | | .117 | | .110 | | −0.007 | |
| Model *p* | .194 | | .041 | | .047 | | .328 | |

*Note.* DSF = Digit Span Forward; DSB = Digit Span Backward; CES-D = Center for Epidemiologic Studies Depression Scale; SVF = semantic verbal fluency (animals). CGG = *FMR1* CGG repeat length. *APOE* any variant = ε2 or ε4 carrier vs. ε3/ε3 reference group. All *p*-values are uncorrected. Analyses conducted in PM carriers with available CGG data (*n* = 46); one PM carrier excluded due to missing CGG data. B = unstandardized coefficient; 95% CI in brackets. CGG repeat length mean-centered; linear and quadratic terms included. Covariates: age, sex, education.
